# Amelioration of pathologic α-synuclein-induced Parkinson’s disease by irisin

**DOI:** 10.1073/pnas.2204835119

**Published:** 2022-08-31

**Authors:** Tae-In Kam, Hyejin Park, Shih-Ching Chou, Jonathan G. Van Vranken, Melanie J. Mittenbühler, Hyeonwoo Kim, Mu A, Yu Ree Choi, Devanik Biswas, Justin Wang, Yu Shin, Alexis Loder, Senthilkumar S. Karuppagounder, Christiane D. Wrann, Valina L. Dawson, Bruce M. Spiegelman, Ted M. Dawson

**Affiliations:** ^a^Neuroregeneration and Stem Cell Programs, Institute for Cell Engineering, Johns Hopkins University School of Medicine, Baltimore, MD 21205;; ^b^Department of Neurology, Johns Hopkins University School of Medicine, Baltimore, MD 21205;; ^c^Department of Cell Biology, Harvard Medical School, Boston, MA, 02215;; ^d^Department of Cancer Biology, Dana-Farber Cancer Institute, Boston, MA, 02215;; ^e^Cardiovascular Research Center, McCance Center for Brain Health, Massachusetts General Hospital and Harvard Medical School, Charlestown, MA, 02129;; ^f^Solomon H. Snyder Department of Neuroscience, Johns Hopkins University School of Medicine, Baltimore, MD 21205;; ^g^Department of Physiology, Johns Hopkins University School of Medicine, Baltimore, MD 21205;; ^h^Department of Pharmacology and Molecular Sciences, Johns Hopkins University School of Medicine, Baltimore, MD 21205

**Keywords:** irisin, Parkinson’s disease, neurodegeneration, synuclein

## Abstract

Physical activity provides clinical benefit in Parkinson’s disease (PD). Irisin is an exercise-induced polypeptide secreted by skeletal muscle that crosses the blood–brain barrier and mediates certain effects of exercise. Here, we show that irisin prevents pathologic α-synuclein (α-syn)-induced neurodegeneration in the α-syn preformed fibril (PFF) mouse model of sporadic PD. Intravenous delivery of irisin via viral vectors following the stereotaxic intrastriatal injection of α-syn PFF cause a reduction in the formation of pathologic α-syn and prevented the loss of dopamine neurons and lowering of striatal dopamine. Irisin also substantially reduced the α-syn PFF-induced motor deficits as assessed behaviorally by the pole and grip strength test. Recombinant sustained irisin treatment of primary cortical neurons attenuated α-syn PFF toxicity by reducing the formation of phosphorylated serine 129 of α-syn and neuronal cell death. Tandem mass spectrometry and biochemical analysis revealed that irisin reduced pathologic α-syn by enhancing endolysosomal degradation of pathologic α-syn. Our findings highlight the potential for therapeutic disease modification of irisin in PD.

## Significance

Physical exercise is thought to have beneficial effects on the symptoms of Parkinson’s disease (PD). Irisin is an exercise-induced myokine released into the circulation. We therefore tested whether irisin itself could have a beneficial effect on pathologic α-synuclein (α-syn) accumulation and concomitant neurodegeneration in PD. Here, we show that irisin prevents the accumulation of pathologic α-syn and neuronal cell death by enhancing endolysosomal degradation of pathologic α-syn. Furthermore, elevation of blood irisin levels in mice prevented neurodegeneration and physiological deficits induced by injection α-syn preformed fibrils. These findings would seem to have translational promise as a disease-modifying therapy for treating PD and other neurodegenerative diseases involving pathologic α-syn.

Parkinson’s disease (PD) is a chronic neurodegenerative disorder characterized by progressive worsening of motor symptoms, including bradykinesia, resting tremor, and rigidity (1, 2). Nonmotor symptoms often precede and accompany the motor symptoms and include autonomic dysfunction and neuropsychiatric sequelae (3). The most notable loss of neurons occurs in the dopaminergic neurons of the substantia nigra pars compacta (SNpc), although neuronal loss also occurs in the locus coeruleus, dorsal raphe nucleus, the dorsal motor nucleus of the vagus, and nucleus basalis of Meynert (4). In addition to neuronal loss, there is accumulation of misfolded pathologic α-synuclein that drives the pathogenesis of PD, including the neuronal dysfunction and the ultimate of neuronal degeneration (5, 6). Current treatments for PD include the replacement of dopamine (DA) via L-DOPA, DA agonists, and other agents to treat the nonmotor symptoms. As the disease progresses, deep brain stimulation and other neurosurgical approaches can be used to treat the side effects of DA replacement therapy. Importantly, these treatments only address the symptomology, and over time there is a progressive decline in normal function. Moreover, there are no treatments that slow the progression or inhibit the underlying drivers of PD pathogenesis. As such, treatments that result in durable arrest of PD symptoms are urgently needed.

Irisin is a small polypeptide that is secreted by skeletal muscle and other tissues into the blood of mice and humans (7, 8). The amino acid sequence is conserved 100% between mice and humans, suggesting a critical, conserved function. Importantly, the expression of irisin and its precursor protein FNDC5 is increased in muscle in response to many forms of exercise, both in rodents and in humans. Irisin levels increase in the blood of humans with exercise training, as determined by tandem mass spectrometry (8). In adipose cells, osteocytes, osteoclasts, and astrocytes integrin αV/β5 is the major functioning receptor for irisin (9, 10).

Physical activity can prevent and ameliorate the symptoms of multiple forms of neurodegeneration, including Alzheimer’s disease (AD) and PD (11–14). Since irisin carries some of the benefits of exercise to adipose tissues, we and others have begun to study the effects of irisin in various models of neurodegeneration. In the earliest study, we showed that elevated expression of FNDC5 in the liver via the use of adenoviral vectors, and presumptive elevations of irisin in the blood, stimulated an “exercise-like” program of gene expression in the hippocampus (15). Moreover, the expression of FNDC5 with these same viral vectors rescued memory deficits in a mouse model of AD (16). Most recently, irisin itself was shown to be the active moiety regulating cognitive function in four separate mouse models. Importantly, elevation of the blood levels of the mature, cleaved irisin using adeno-associated virus (AAV) was sufficient to improve cognitive function and reduce neuroinflammation in two distinct models of AD (9). Furthermore, irisin itself crossed the blood–brain barrier (BBB), at least when the protein was produced from the liver with these AAV vectors.

In the current study, we examine the effects of irisin on the pathophysiology of PD, using the α-synuclein preformed fibril (α-syn PFF) seeding model in vitro and in vivo. Pathologic α-syn is thought to spread “prion-like” in the brains of PD patients and certain other neurological disorders, where they cause neuronal death and dysfunction. We show here that irisin has powerful effects in preventing both the accumulation of pathologic α-syn and neuronal cell death in primary cell culture. Furthermore, elevation of blood irisin levels in mice normalizes the histological manifestations in the SNpc and the PD-like symptomology involving movement and grip strength induced by intrastriatal injection of α-syn PFF. Together, these data suggest the potential therapeutic value of irisin in PD and other neurodegenerative states that involve α-syn.

## Results

### Irisin Prevents the Formation of Pathologic α-Syn and Protects Neurons against α-Syn PFF-Induced Neurotoxicity.

**Fig. 1.**
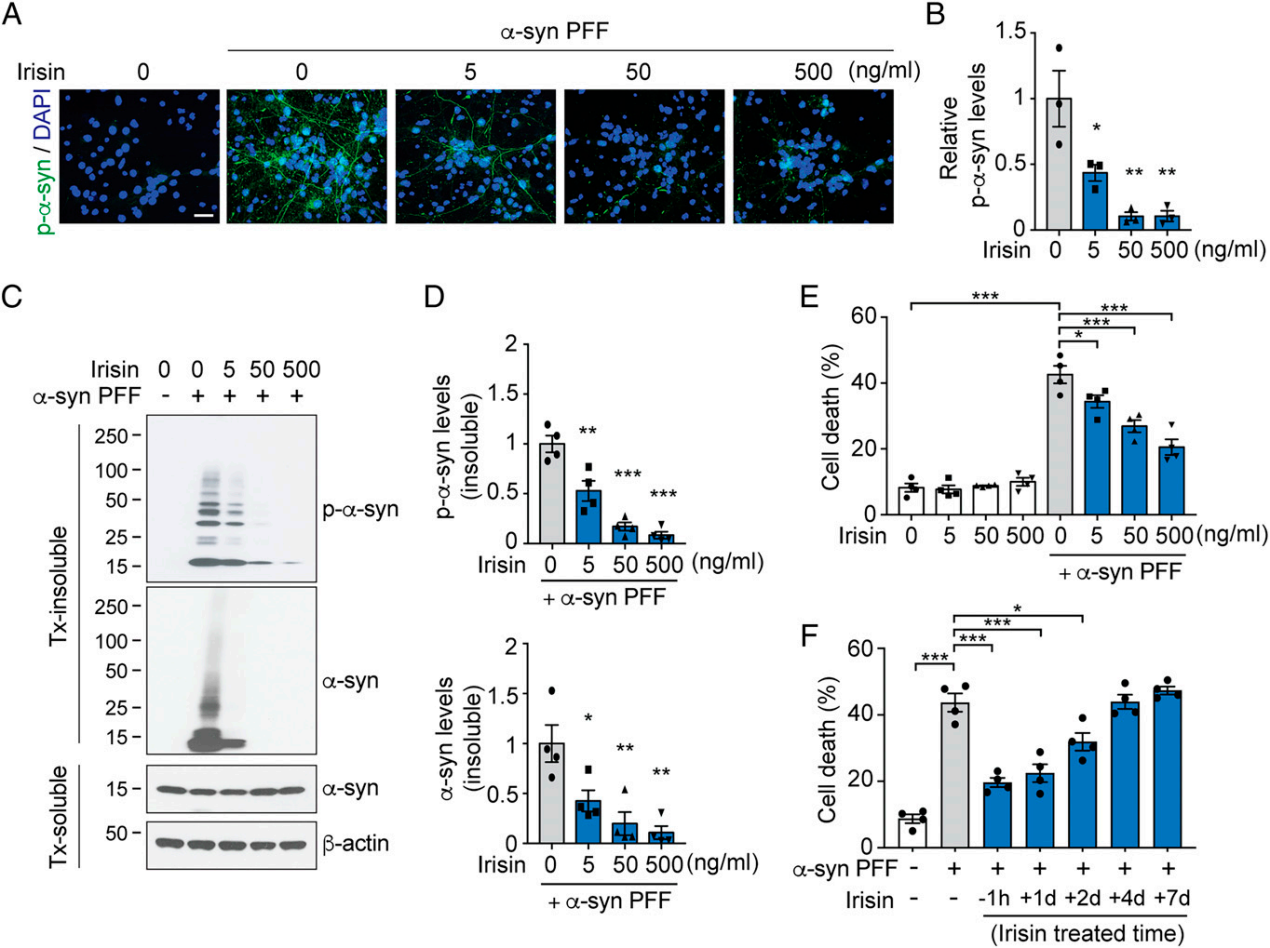
Irisin protects neurons against α-syn PFF neurotoxicity. (*A*) Representative images of pS129-α-syn (green) in primary cortical neurons preincubated for 1 h followed by sustained treatment with indicated concentration of irisin, and further incubated with α-syn PFF (1 μg/mL) for 7 d. DAPI (blue) was used for nuclei staining. (Scale bar, 20 μm.) (*B*) Quantification of p-α-syn signals in (*A*) normalized with DAPI. Bars represent mean ± SEM. One-way ANOVA followed by Tukey’s post hoc test (*n* = 3). (*C*) Representative immunoblots of pS129-α-syn and α-syn in the Triton X-100-soluble and insoluble fraction from primary cortical neurons preincubated for 1 h followed by sustained treatment with indicated concentration of irisin followed by incubation with α-syn PFF for 7 d. (*D*) Quantification of levels of pS129-α-syn and α-syn in the Triton X-100-insoluble fraction normalized to β-actin shown in (*C*). Bars represent mean ± SEM. One-way ANOVA followed by Tukey’s post hoc test (*n* = 4). (*E*) Cell death assay quantified from Hoechst and propidium iodide (PI) staining in primary cortical neurons treated for 1 h followed by sustained treatment with indicated concentration of irisin and further incubated with α-syn PFF (5 μg/mL) for 14 d. Bars represent mean ± SEM. Two-way ANOVA followed by Tukey’s post hoc test (*n* = 4). (*F*) Cell death assay quantified from Hoechst and propidium iodide (PI) staining in primary cortical neurons preincubated 1 h followed by sustained treatment with irisin (50 ng/mL) and further incubated with α-syn PFF (5 μg/mL) for 14 d as well as delayed treatment (1 d, 2 d, 4 d, and 7 d) after α-syn PFF treatment. Bars represent mean ± SEM. One-way ANOVA followed by Tukey’s post hoc test (*n* = 4). *P < 0.05, **P < 0.005, ***P < 0.0005.

α-Syn PFF administration to cultured primary cortical neurons induces endogenous α-syn to misfold and become toxic to those cells (17, 18). This transformation can be monitored by the phosphorylation of α-syn serine 129 (pSer129-α-syn) (19). One hour pre- and continuous treatment of cortical neurons for the duration of the α-syn PFF treatment with 5 ng/mL of irisin significantly reduced the levels of p-α-syn, and 50 and 500 ng/mL of irisin essentially prevented the formation of p-α-syn as determined via immunocytochemistry (Fig. 1 *A* and *B*) and immunoblot analysis (Fig. 1 *C* and *D*) 7 d after the α-syn PFF administration. Irisin also prevented the accumulation of Triton X-100 (Tx)-insoluble p-α-syn and α-syn (Fig. 1 *C* and *D*). One hour pre- and sustained treatment of cortical neurons with 5, 50, and 500 ng/mL of irisin prevented the death of cortical neurons induced by α-syn PFF as assessed 14 d after treatment with α-syn PFF (Fig. 1*E*). In addition to the 1 h irisin pre- and sustained treatment, a delay in the sustained irisin treatment by 1 or 2 d after administration of α-syn PFF was able prevent the death of cortical neurons (Fig. 1*F*). When the irisin treatment was delayed to 4 and 7 d post α-syn PFF treatment, irisin had no effect on α-syn PFF-induced cell death (Fig. 1*F*). Taken together, these data indicated that irisin prevents the formation of pathologic α-syn and protects neurons against α-syn PFF-induced neurotoxicity.

### Irisin Prevents the DA Neuronal Loss in the Intrastriatal α-Syn PFF Mouse Model.

**Fig. 2.**
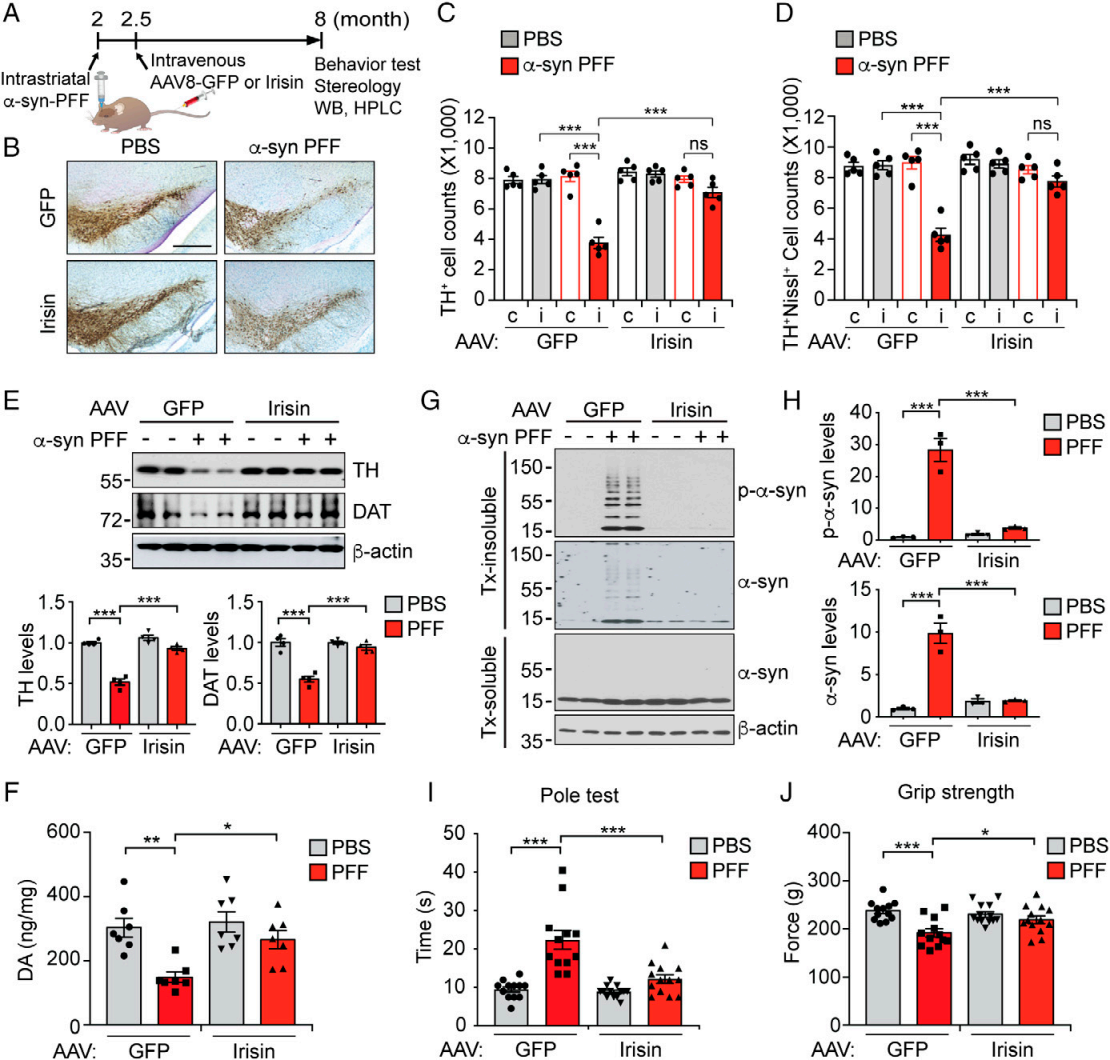
Irisin protects α-syn PFF-induced pathology in vivo. (*A*) Diagram of in vivo experiments. Two-month-old WT mice were injected with PBS or α-syn PFF (5 μg/mouse) into the striatum. Two weeks after α-syn PFF injection, the mice were injected with AAV8-GFP or AAV8-Irisin-FLAG (1E10 G.C./mouse) via the tail vein. The mice were subjected to behavioral test (pole test and grip strength test), stereology and biochemical analysis 6 mo after α-syn PFF injection. (*B*) Representative TH and Nissl staining of SNpc DA neurons of PBS or α-syn PFF injected mice treated with AAV-GFP or AAV-Irisin. (Scale bars, 400 μm.) (*C*, *D*) Stereological counts of (*C*) TH^+^ and (*D*) TH^+^/Nissl^+^ cells. Data are mean ± SEM. Two-way ANOVA followed by Tukey’s post hoc test (*n* = 5 mice per group). i = ipsilateral, c = contralateral. (*E*) Representative immunoblots and quantification of TH and DAT in the ipsilateral striatum of injected mice. Bars represent the mean ± SEM. Two-way ANOVA followed by Tukey’s post hoc test (*n* = 4). (*F*) DA concentrations in the striatum of PBS or α-syn PFF injected mice treated with AAV-GFP or AAV-Irisin determined by HPLC. Bars represent mean ± SEM. Two-way ANOVA followed by Tukey’s post hoc test. (*n* = 7 mice per group). (*G*) Representative immunoblots of pS129-α-syn and α-syn in the detergent-soluble and insoluble fraction from the SNpc of injected mice. (*H*) Quantification of pS129-α-syn and α-syn levels in the detergent-insoluble fraction normalized to β-actin. Bars represent mean ± SEM. Two-way ANOVA followed by Tukey’s post hoc test (*n* = 3). (*I*, *J*) Pole test (*I*) and grip strength (*J*) test were performed 6 mo after PBS or α-syn PFF injection. Data are the mean ± SEM *P < 0.05, ***P < 0.0005, two-way ANOVA followed by Tukey’s post hoc test (*n* = 12–13 mice per group). *P < 0.05, **P < 0.005, ***P < 0.0005.

To determine whether irisin can prevent pathologic α-syn-induced degeneration in vivo, α-syn PFF were stereotaxically injected into the striatum of mice (17, 18). The protective role of irisin was evaluated by use of an AAV-irisin vector (AAV8) that is mainly taken up by the liver after tail vein injection and elevates circulating irisin (9). Two weeks after the intrastriatal α-syn PFF injection, mice were either injected via the tail vein with AAV8-irisin-FLAG or as a control, AAV8-GFP (Fig. 2*A*). Prior studies indicate that this route of administration of cleaved, mature irisin provides sufficient brain levels of irisin to reduce the pathology in two models of AD (9). Importantly, this vector and does not infect and express within the brain (9). 5.5 mo after the tail vein injection of AAV8-irisin-FLAG, we observed that irisin-FLAG was significantly elevated in the plasma and liver in both intrastriatal phosphate-buffered saline (PBS) and α-syn PFF injected mice (*SI Appendix*, Fig. S1 *A* and *B*). Intravenous injection of irisin-His peptide (1 mg/kg) in mice, led to a significant elevation of irisin-His in plasma and brain, indicating that exogenous irisin is capable of increasing irisin levels in the brain by crossing the blood brain barrier (*SI Appendix*, Fig. S1 *C* and *D*).

The pathogenic spread of α-syn is detectable in the substantia nigra 1 mo after the intrastriatal α-syn PFF injection (17). As previously described (18, 20), there was an approximate 50% loss of DA neurons 6 mo after intrastriatal injection of α-syn PFF in wild-type (WT) mice as assessed via nonbiased stereologic counts of tyrosine hydroxylase (TH) and Nissl-stained neurons (Fig. 2 *B*–*D*). AAV8-Irisin injection reduced the loss of DA neurons when compared to AAV8-GFP injected mice (a 60% loss as compared to only a 25% loss in the presence of irisin) (Fig. 2 *B*–*D*). The rescue effect of irisin on TH and DAT protein levels measured by immunoblot were even larger. The levels of TH and dopamine transporter (DAT) were decreased in α-syn PFF-injected mice by 49% and 45%, respectively, and this reduction was reduced by AAV8-Irisin to a level of 6% and 6%, respectively (Fig. 2*E*). In addition to DA neuronal loss, TH fiber density was reduced in striatum of α-syn PFF-injected mice with AAV8-GFP injection, but not with AAV8-Irisin injection (*SI Appendix*, Fig. S2 *A* and *B*). High-performance liquid chromatography (HPLC) revealed that there was a reduction in striatal DA and its metabolites, 3,4-dihydroxyphenylacetic acid (DOPAC), homovanillic acid (HVA), and 3-methoxytyramine (3-MT) in α-syn PFF-injected mice. This loss of byproducts of dopamine and its metabolites were blocked by 87%, 95%, 72% and 70%, respectively in AAV8-Irisin injected mice (Fig. 2*F* and *SI Appendix*, Fig. S2 *C*–*E*). DA turnover was increased in striatal α-syn PFF injected mice with AAV8-GFP injection, while these effects were suppressed in AAV8-irisin injected mice (*SI Appendix*, Fig. S2 *F* and *G*). Importantly, AAV8-irisin also blocked the accumulation of insoluble pathologic p-α-syn and α-syn compared to AAV8-GFP treated mice, while having no effects on soluble α-syn monomer levels (Fig. 2 *G* and *H*). Furthermore, AAV8-irisin largely prevented the behavioral deficits induced by α-syn PFF-induced as determined by the pole test (Fig. 2*I*) and grip strength test (Fig. 2*K* and *SI Appendix*, Fig. S2*H*). Taken together, these results indicate that irisin prevents the damage and loss of DA neurons and, consequently, the neurobehavioral deficits induced by striatal α-syn PFFs.

### Irisin Inhibits the Internalization and Propagation of α-Syn.

**Fig. 3.**
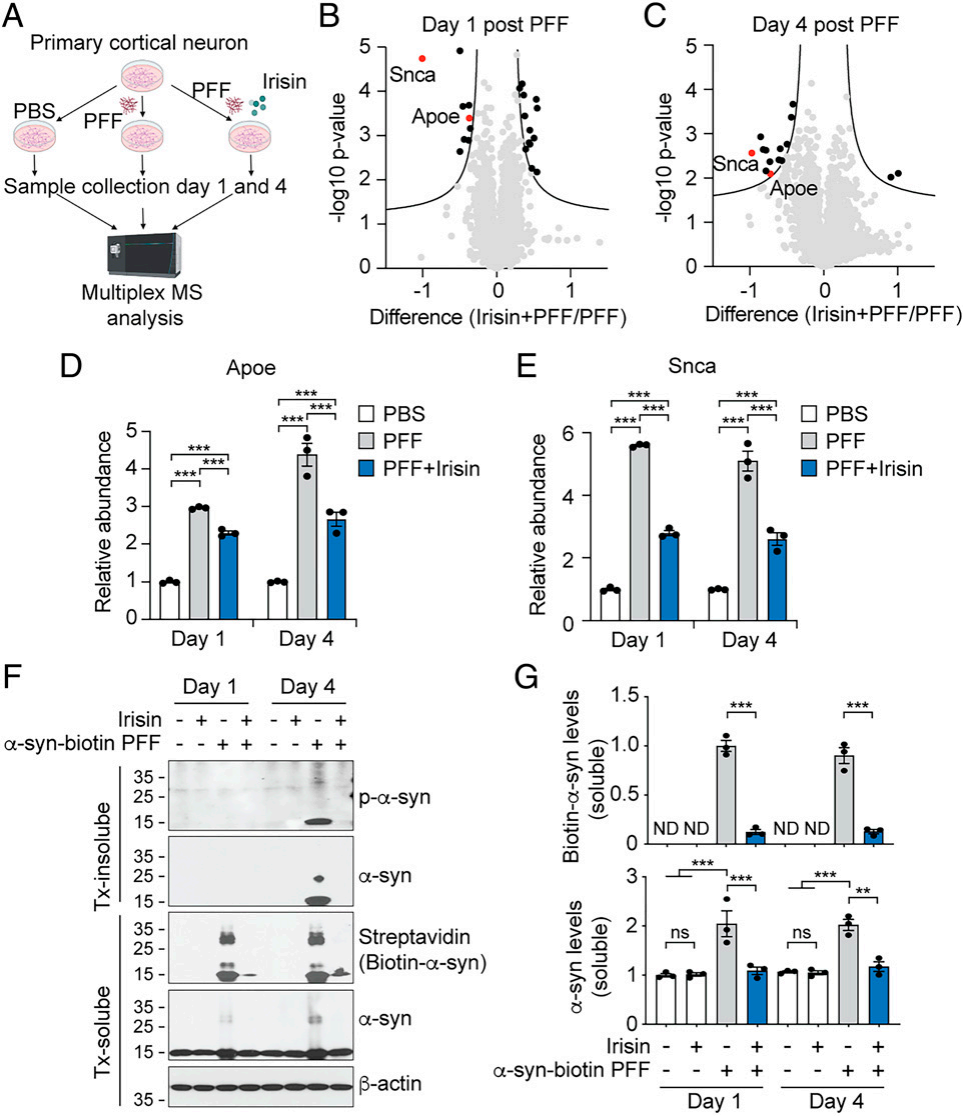
Irisin reduces the α-syn levels. (*A*) Schematic diagram of proteomic analysis. (*B*, *C*) Volcano plot of protein alterations. The proteins quantified from primary cortical neurons with or without preincubation of irisin (50 ng/mL) and further incubated with α-syn PFF (1 μg/mL) for (*B*) one or (*C*) 4 d were analyzed for differentially expressed proteins in PFF- and irisin-treated cells. The cutoff used to select differentially expressed proteins was q-value < 0.05. (*D*, *E*) Relative protein levels of (*D*) ApoE and (*E*) Snca in primary cortical neurons 1 and 4 d after PBS, α-syn PFF, or α-syn PFF with irisin administration analyzed by mass spec. Bars represent mean ± SEM. Two-way ANOVA followed by Tukey’s post hoc test (*n* = 3). (*F*) Representative immunoblots of pS129-α-syn, α-syn and α-syn-biotin in the detergent- insoluble and soluble fraction from cortical neurons 1 and 4 d after treatment. (*G*) Quantification of α-syn-biotin and α-syn levels in the detergent-soluble fraction normalized to β-actin. Bars represent mean ± SEM. Two-way ANOVA followed by Tukey’s post hoc test (*n* = 3). *P < 0.05, **P < 0.005, ***P < 0.0005. ND, not determined; ns, not significant.

To determine the molecular pathways by which irisin might prevent α-syn PFF-induced neurodegeneration, the proteomes from primary cultured cortical neurons treated with α-syn PFF for 1 or 4 d in the absence and presence of irisin were analyzed by liquid chromatography–tandem mass spectrometry (LC-MS/MS) (Fig. 3*A*). Statistical analysis of the quantified proteins showed that 2 and 26 proteins were differentially regulated by α-syn PFF at 1 and 4 d after treatment, respectively (*SI Appendix*, Fig. S3 *A* and *B* and Dataset S1). Among them, 100% and 34.6% of proteins 1 and 4 d after α-syn PFF treatment were counter regulated by irisin (Fig. 3 *B* and *C*). Of note, irisin treatment significantly changed the abundance of 22 and 15 proteins 1 and 4 d after α-syn PFF treatment when compared to α-syn PFF-treated neurons only (Fig. 3 *B* and *C*). α-Syn PFF treatment significantly up-regulated the ApoE protein (Fig. 3*D*), whose ε4 genotype in humans regulates α-syn pathology (21) and is associated with an increased risk of dementia in PD (22, 23) and AD (24). Irisin significantly down-regulated ApoE (Fig. 3*D*). Importantly, the α-syn protein itself, which increased after α-syn PFF administration showed a decrease following irisin treatment 1 and 4 d later (Fig. 3*E*). The levels of α-syn in Tx-soluble and Tx-insoluble fractions after treatment of cortical neurons with biotin-labeled α-syn PFF (α-syn-biotin PFF) and irisin were measured. Prior experiments have shown that α-syn-biotin enters neurons and templates endogenous α-syn to misfold and become pathogenic, in a manner similar to unlabeled α-syn PFF (20). One hour pre- and continuous treatment of cortical neurons with 50 ng/mL of irisin significantly reduced the levels of α-syn-biotin PFF in both the Tx-soluble fraction after 1 or 4 d and in the Tx-insoluble fraction 4 d after α-syn PFF administration (Fig. 3 *F* and *G*). Endogenous α-syn levels paralleled the changes in α-syn-biotin (Fig. 3 *F* and *G*). Pathologic formation of p-α-syn in the Tx-insoluble fraction starts 4 d after α-syn PFF administration (17), which was prevented by irisin (Fig. 3*F*). Taken together, these data suggest that irisin may prevent the intracellular accumulation of a pSer129-positive pathologic form of α-syn by decreasing its internalization and aggregation.

### Irisin Enhances the Lysosomal Degradation of α-Syn PFF.

**Fig. 4.**
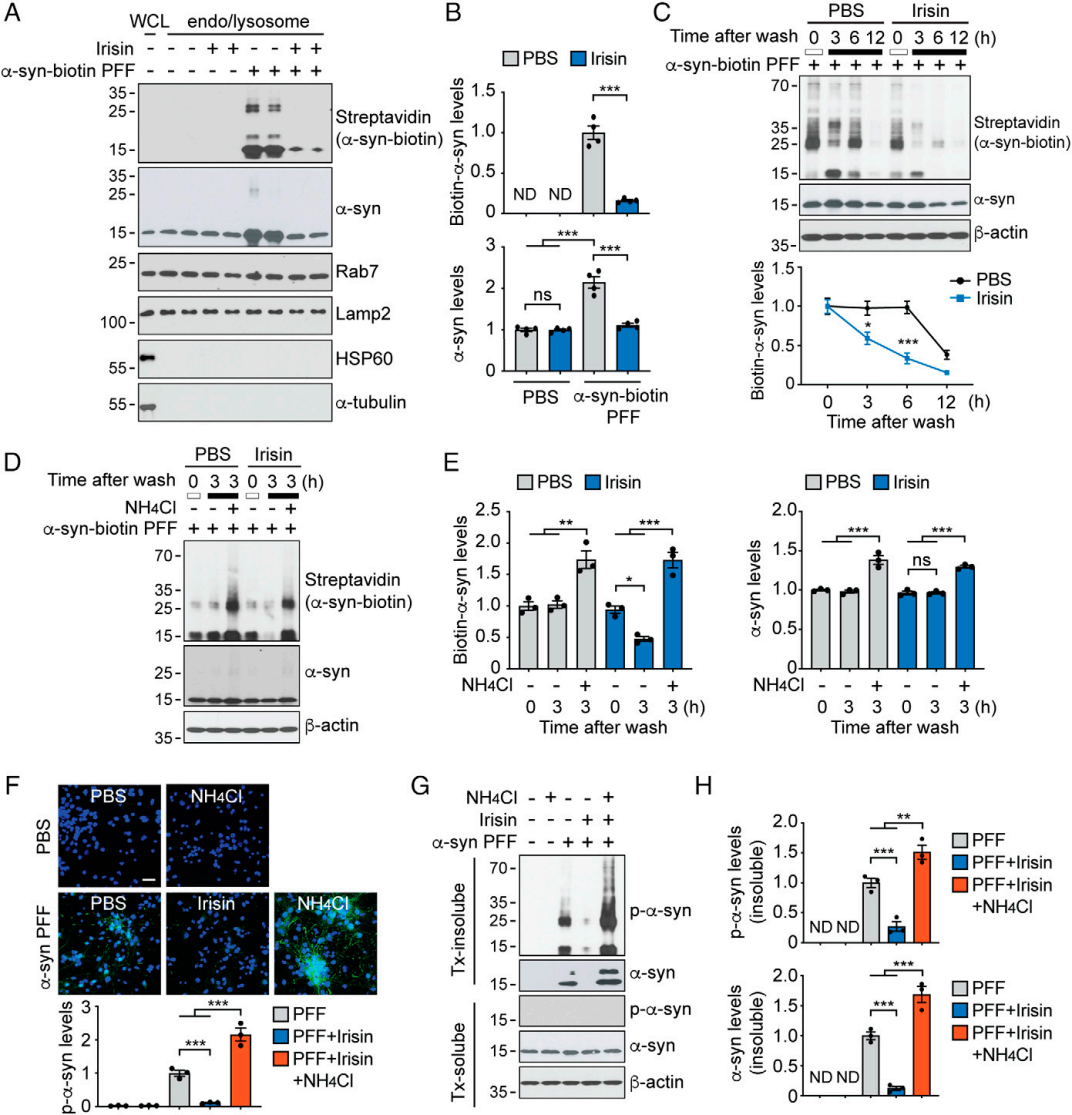
Irisin increases the degradation of pathologic α-syn. (*A*, *B*) Primary cortical neurons from WT embryos were pretreated with 50 ng/mL Irisin for 1 h and further incubated with biotin-conjugated α-syn PFF (1 μg/mL) for 24 h. The levels of α-syn-biotin and α-syn in the endolysosome-enriched fraction were determined by immunoblotting using anti-streptavidin and an anti-α-syn antibody, respectively. Rab7 is a marker for endosome, Lamp2 is a marker for lysosome, HSP60 is a marker for mitochondria, and α-tubulin is a marker for cytoplasm. Bars represent mean ± SEM. Two-way ANOVA followed by Tukey’s post hoc test (*n* = 4). (*C*) Irisin promotes intracellular degradation of propagated α-syn PFF. Primary cortical neurons were pretreated with 50 ng/mL Irisin for 1 h and further incubated with biotin-conjugated α-syn PFF (1 μg/mL) in the presence of 50 ng/mL irisin for 12 h followed by media replacement with 50 ng/mL irisin not containing α-syn PFF. Intracellular biotin-conjugated α-syn PFF levels were determined by immunoblotting using anti-streptavidin antibody 3, 6, and 12 h after changing to fresh medium containing 50 ng/mL irisin. Graph represents mean ± SEM. Two-way ANOVA followed by Tukey’s post hoc test (*n* = 3). (*D*, *E*) Propagated α-syn PFF is degraded by the lysosome. Primary cortical neurons were pretreated with 50 ng/mL Irisin for 1 h and further incubated with biotin-conjugated α-syn PFF (1 μg/mL) for 12 h, followed by the fresh medium or medium containing NH_4_Cl was replaced for 3 h. The levels of α-syn-biotin and α-syn were determined by immunoblotting using anti-streptavidin and α-syn antibodies, respectively. Graph represents mean ± SEM. Two-way ANOVA followed by Tukey’s post hoc test (*n* = 3). (*F*) Representative microscopic images of pS129-α-syn (green) in primary cortical neurons treated with α-syn PFF (1 μg/mL) for 4 d. Two days after α-syn PFF treatment, irisin and NH_4_Cl were incubated for 2 d. DAPI (blue) is used for nuclei staining. (Scale bar, 20 μm.) Quantification of p-α-syn signals was normalized with DAPI. Bars represent mean ± SEM. One-way ANOVA followed by Tukey’s post hoc test (*n* = 3). (*G*) Representative immunoblots of pS129-α-syn and α-syn in the detergent-soluble and insoluble fraction from primary cortical neurons incubated with α-syn PFF for 4 d followed by posttreated with irisin and NH_4_Cl for 2 d. (*H*) Quantification of pS129-α-syn and α-syn levels in the detergent-insoluble fraction normalized to β-actin. Bars represent mean ± SEM. One-way ANOVA followed by Tukey’s post hoc test (*n* = 4). *P < 0.05, **P < 0.005, ***P < 0.0005. ND, not determined; ns, not significant.

α-Syn PFF are taken up into neurons via receptor mediated endocytosis, micropinocytosis or tunneling nanotubes (20, 25–27) where they end up in the endolysosomes. Here, it is thought that they template endogenous monomeric α-syn to form pathologic α-syn, thus initiating a neurodegenerative cascade (28–30). We therefore asked whether irisin might inhibit the intracellular accumulation of α-syn by regulating endolysosomal degradation of α-syn. α-Syn PFF levels in the endolysosomes-containing fraction after treatment of primary cortical neurons with α-syn-biotin PFF and irisin were measured. One hour pre- and continuous treatment of cortical neurons with 50 ng/mL of irisin significantly reduced α-syn-biotin PFF levels in the endolysosomes-containing fraction (Fig. 4 *A* and *B*). To determine whether internalized α-syn-biotin PFF is degraded via the lysosomal system or through the ubiquitin proteasome system, the effect of the well-known lysosomal inhibitor NH_4_Cl or the proteasome inhibitor MG132 affected the degradation of α-syn in the absence or presence of irisin was evaluated. In the absence of irisin, NH_4_Cl prevented the degradation of α-syn-biotin PFF, while MG132 had no effect (*SI Appendix*, Fig. S4 *A* and *B*). To detect the endolysosomal degradation of internalized α-syn, primary cultured cortical neurons were pretreated with 50 ng/mL irisin for 1 h and further incubated with 50 ng/mL irisin and α-syn-biotin PFF (1 μg/mL) for 12 h. In this experiment, the medium was replaced with irisin and no α-syn-biotin PFF. This irisin treatment significantly reduced α-syn-biotin PFF levels in cortical neurons at 3 h and 6 h after the media replacement of cortical neurons (Fig. 4*C*). This reduction in the levels of α-syn-biotin PFF by irisin treatments was inhibited by NH_4_Cl (Fig. 4 *D* and *E*) suggesting that irisin reduces pathologic α-syn by enhancing the pathway of lysosomal-mediated degradation. To confirm that irisin-induced lysosomal degradation of internalized α-syn PFF reduces pathologic α-syn, the effect of the irisin in the presence and absence of NH_4_Cl on the formation of p-α-syn was monitored (Fig. 4 *F*–*H*). Primary cultured cortical neurons were treated with α-syn PFF for 2 d followed by treatment with 50 ng/mL of irisin in the presence and absence of a low dose of NH_4_Cl, which had previously been shown to exhibit minimal neurotoxicity (31). Posttreatment with irisin 2 d after α-syn PFF administration significantly reduced the levels of p-α-syn, while coadministration of NH_4_Cl prevented the reduction of p-α-syn by irisin (Fig. 4 *F*–*H*). Taken together, these results suggest that irisin, at least in part, prevented the pathologic transmission of α-syn by promoting the endolysosomal degradation of α-syn PFF.

## Discussion

The major phenotypic conclusion of this paper is that irisin prevents the degeneration of DA neurons and thereby reduces the motor deficits induced by pathologic α-syn. Mechanistically, it appears that irisin reduces the level of pathologic α-syn at least in part, through increasing the lysosomal degradation of pathologic α-syn.

These studies were motivated by the strong evidence that exercise is beneficial in PD (32–34). The molecular mechanisms accounting for beneficial effects of exercise are not fully understood. We show here that irisin protects against pathologic α-syn, by enhancing its lysosomal degradation. Consistent with this notion, other reports indicate that irisin can enhance autophagic lysosomal activity (35–38). On the other hand, this may not be the complete mechanism. In AD, the effects of irisin seem to be mediated, in part, through an increase in brain-derived neurotrophic factor (BDNF) (15) and/or inhibition of glial activation (9). Irisin has also been shown to offer protection in the 1-methyl-4-phenyl-1,2,3,6-tetrahydropyridine (MPTP) intoxication model of PD through possibly enhancing the import of bone marrow-derived stem cells (39). Therefore, the effect of irisin may be diverse. However, our demonstration that irisin reduces pathologic α-syn is particularly relevant to the pathogenesis of PD and related α-synucleinopathies since pathologic α-syn appears to be the major pathogenic driver of these disorders.

Irisin is a muscle-derived molecule whose levels increase in response to exercise. Here, we sought to address a critical clinical issue with a neurodegenerative disease like PD. PD starts with mild symptoms and often proceeds slowly over years to produce major neurological deficits in motor activity and cognition. We asked whether, after the onset of biochemical manifestations induced by intrastriatal injection of α-syn PFF, irisin could slow or reverse disease. Irisin itself has a short half-life in the blood, and hence we used AAV expression of a transgene for cleaved irisin administered via tail vein injection. The AAV-irisin construct was injected 2 wk after the intrastriatal α-syn PFF injection. Despite this delay, irisin protects against the pathogenic cascade induced by α-syn PFF injection. In these experiments pathologic α-syn has spread to substantia nigra 1 mo after the intrastriatal injection of α-syn PFF (17) at a time when irisin, using a similar injection protocol of AAV-irisin, has been shown to be expressed at sufficient brain levels (9). This suggests that the first steps of α-syn PFF activation have already occurred. However, it is difficult to know exactly how long between the α-syn PFF injection and spread of pathologic α-syn and irisin administration, that irisin acts. As such, whether irisin halts the spread of the disease or reverses the disease is not known. It will be important for any future human therapy to determine whether irisin can arrest the progression of experimental PD after neurological symptoms have started and to determine the effects of irisin in other PD models. Nevertheless, since irisin treatment was begun well after the pathogenic cascade was initiated by the α-syn PFF injection, there is considerable promise that it might be developed as a disease-modifying therapy for the treatment of PD. Therefore, optimization of irisin delivery as a biologic therapy holds promise for the treatment of PD and other neurodegenerative disorders.

## Materials and Methods

### Animal.

C57BL/6 WT mice used in experiments were purchased from the Jackson Laboratories (Bar Harbor, ME, USA). All animal experiments were approved by Johns Hopkins University Animal Care and Use Committee and performed according to the NIH Guide for the Care and Use of Experimental Animals.

### Preparation of α-Syn PFF.

Monomeric mouse α-syn recombinant proteins were prepared as described previously (17), The Toxineraser endotoxin removal kit (GeneScript) was used to remove endotoxins. For generation of α-syn PFF, α-syn proteins were constantly agitated with a thermomixer (1,000 rpm at 37 °C) (Eppendorf) for 7 d, followed by sonication for 30 s (10% amplitude) with 0.5 s pulse on and off cycle (Branson Digital Sonifier) before use. For generation of α-syn-biotin PFF, biotin was conjugated to monomeric α-syn with biotin (2–3 molar ratio) using EZ-link Sulfo-NHS-LC-Biotin (Thermo Scientific). α-Syn-biotin PFF was prepared in a manner similar to α-syn PFF.

### Preparation of Irisin and AAV-Irisin.

Recombinant protein was prepared in mammalian cells as previously described (10). The Irisin-flag construct was prepared as previously described (9). The pENN.AAV.CB7.CI.pm20d1flag.WPRE.rBG vector (Addgene plasmid #132682) replaced pm20d1flag using the PstI/HindIII restriction enzymes was used for cloning of the N-terminal part of mouse FNDC5 (signal peptide, amino acid residues 1–28) and irisin ORF plus flag-tag. The correct insertion of the signal peptide of mouse FNDC5 and irisin ORF was confirmed by Sanger sequencing. Packaging into the AAV (serotype 8) was performed at the Penn Vector Core. AAV8-GFP (pENN.AAV.CB7.CI.eGFP.WPRE.rBG) was used as control, generated by the Penn Vector Core, and obtained from Addgene (Addgene #105542).

### Stereotaxic Injection of α-Syn PFF and Intravenous Injection of AAV-Irisin.

Two-month-old WT mice were positioned on a stereotaxic instrument after anesthetizing with 100 mg/kg of ketamine and 20 mg/kg of xylazine. α-Syn PFF (5 μg/2 μL) was injected into a right striatum (anteroposterior [AP] = +2.0 mm, mediolateral [ML] = ±2.0 mm, dorsoventral [DV] = +2.8 mm from bregma). After completion of injection with a speed of 0.4 μL/min and maintaining a needle for additional 5 min, postsurgical care was provided. Two weeks after α-syn PFF injection, AAV8-GFP or AAV8-Irisin-FLAG (100 μL of 1 × 10^10^ GC per mouse) was injected into the tail vein. Six months after injection, behavioral tests and biochemical analyses were performed. Blood was collected, and the plasma fraction was collected by centrifugation. The irisin-FLAG levels in plasma were determined by ELISA as previously described (9). Brains were immediately dissected and frozen at −80 °C for biochemical analysis. For histological studies, mice were perfused with ice-cold PBS and 4% paraformaldehyde (PFA) and brains were postfixed in 4% PFA, followed by cryoprotection in 30% sucrose solution.

### Measurement of Irisin Accumulation in the Brain.

C57BL/6 mice were intravenously injected with purified irisin-His (1 mg/kg) for 1 h. After collection of plasma, mice were perfused with PBS and brains were immediately removed. Levels of irisin-His in plasma and brain lysates were determined by the His tag ELISA detection kit (GenScript) according to manufacturer specifications.

### Behavioral Tests.

Pole test and grip strength test were performed 6 mo after α-syn PFF injection in a double blinded manner with regard to treatment conditions and randomly allocated to groups. For the pole test, a 75 cm long with a 9 mm diameter-sized metal rod wrapped with bandage gauze was used. Mice were trained in three test trials for 2 d. In the actual test, the time to turn and total time to place all four paws on the base were measured after placing the mice 7.5 cm from the top of the pole. The maximum cutoff time was 60 s. The pole was cleaned using 70% ethanol after finishing each trial. For the grip strength test, mice were placed onto a metal grid of the apparatus (Bioseb). Maximal peak force, the peak tension before the mouse loses grip when the tail was gently pulled, with either forelimb or both limbs (fore and hindlimb) were measured in grams (g).

### Primary Neuronal Culture and Treatment.

Primary cortical neurons from WT embryos at embryonic day 16 were cultured as previously described (40). Cultured neurons were treated with 5-Fluorodeoxyuridine (5-FDU) (MP Biomedicals) at a final concentration of 10 μM only one time. Therefore, half the medium was exchanged with fresh neurobasal medium containing 20 µM 5-FDU once 24 h after seeding. Cells were then maintained in neurobasal media containing B-27, 0.5 mM L-glutamine, penicillin, and streptomycin (Invitrogen). Half the neurobasal medium was changed every 3-4 d, and therefore the 5-FDU was diluted upon subsequent medium changes. For Irisin treatments, the protein was added to the culture medium to the indicated final concentrations for 1 h on 7 d in vitro (7 DIV). After 1 h preincubation with irisin, half of the cell culture medium was replaced with fresh medium containing α-syn PFFs plus irisin. For the subsequent irisin treatments, half the cell culture medium was replaced with fresh medium alone or medium containing irisin every 3–4 d. Sequential extraction of Triton X-100-soluble and insoluble α-syn was performed as described previously (41). Neuronal lysates were prepared in Triton lysis buffer (50 mM Tris, [pH 7.6] 150 mM NaCl, 1% Triton X-100, phosphatase inhibitor mixture I and II [Sigma-Aldrich], and complete protease inhibitor mixture [Roche]). The Triton-soluble fraction was collected from the supernatants after sonication followed by centrifugation at 100,000 × *g* for 30 min at 4 °C. The remaining pellets were washed in Triton lysis buffer and resuspended into sodium dodecyl sulfate (SDS) lysis buffer (50 mM Tris, [pH 7.6] 150 mM NaCl, 2% SDS, phosphatase inhibitor mixture I and II [Sigma-Aldrich], and complete protease inhibitor mixture [Roche]), sonicated, and centrifuged at 100,000 × *g* for 30 min at room temperature. The supernatants were used as the Triton-insoluble fraction.

### Cell Death Measurement.

Primary cortical neurons were treated with α-syn PFF (5 μg/mL) in the presence or absence of irisin for 14 d. Neurons were stained with Hoechst 33342 (7 µM) and propidium iodide (PI) (2 µM) (Invitrogen). Images were taken by microscope and dead cells were automatically counted by Axiovision 4.6 software (Carl Zeiss).

### Tissue Lysate and Western Blot Analysis.

Brain tissues were homogenized using a Diax 900 homogenizer (Sigma-Aldrich) in lysis buffer (50 mM Tris × HCl [pH 7.4], 150 mM NaCl, 1 mM ethylenediaminetetraacetic acid (EDTA), 1% Triton X-100, 0.5% SDS, 0.5% sodium-deoxycholate, phosphatase inhibitor mixture I and II [Sigma-Aldrich], and protease inhibitor mixture [Roche]). After incubation at 4 °C for 30 min for complete lysis, the homogenates were centrifuged at 15,000 × *g* for 20 min and the supernatants were collected. Protein concentration was determined using the BCA assay (Pierce). For Western blot analysis, samples in loading buffer were separated by SDS-PAGE gel electrophoresis. The proteins were transferred onto nitrocellulose membranes, blocked with 5% nonfat milk in TBS-T (Tris-buffered saline with 0.1% Tween-20), and then subjected to immunoblotting using indicated primary antibodies (*SI Appendix*, Table S1) with HRP-conjugated secondary antibodies (Cell Signaling).

### Endolysosome Enrichment.

Internalized α-syn-biotin PFF was detected in endolysosome-containing fractions prepared as previously described (20). α-Syn-biotin PFF-treated primary cortical neurons were incubated with trypsin to remove the membrane-bound α-syn-biotin PFF. Neurons were lysed using a syringe in lysis buffer (250 mM sucrose, 50 mM Tris × HCl [pH 7.4], 5 mM MgCl_2_, 1 mM EDTA, 1 mM EGTA), and a complete protease inhibitor mixture (Roche). Endolysosome-containing fractions were obtained by sequential centrifugation at 1,000 × *g* for 10 min, 16,000 × *g* for 20 min, and 100,000 × *g* for 60 min at 4 °C. The endolysosomes contained in the remaining pellet was centrifuged again at 100,000 × *g* for 60 min after washing with ice-cold PBS. The resuspended pellet in lysis buffer was used for endolysosome experiments.

### Immunohistochemistry and Immunofluorescence.

For immunohistochemistry (IHC), serial brains sections were prepared with 40-μm thickness. After blocking with 10% goat serum in PBS with 0.2% Triton X-100, free-floating sections were incubated with TH antibodies and biotin-conjugated secondary antibody. Sections were developed by adding ABC reagent (Vector Laboratories) and SigmaFast DAB peroxidase substrate (Sigma-Aldrich), followed by counterstaining with Nissl (0.09% thionin). Both TH- and Nissl-positive DA neurons in the substantia nigra were counted in randomly allocated groups in a double blinded manner with respect to the treatment condition and using a computer-assisted image analysis system consisting of an Axiophot photomicroscope (Carl Zeiss) equipped with a computer controlled motorized stage (Ludl Electronics), a Hitachi HV C20 camera, and Stereo Investigator software (MicroBright-Field) (42). For immunofluorescence analysis in primary cultures, Ser129 p-α-syn antibodies were incubated followed by Alexa-fluor 488-conjugated secondary antibodies (Invitrogen). The images were taken by confocal microscopy (LSM710, Carl Zeiss) and processed by the Zen software (Carl Zeiss). The signal intensity was quantified using ImageJ software.

### Dopamine and Derivatives Measurement Using HPLC.

The striatum dissected from the brain were sonicated in ice-cold perchloric acid (0.01 mM) containing 0.01% EDTA. The homogenates were centrifuged at 15,000 × *g* for 30 min at 4 °C and the debris in supernatants were removed using a 0.2 μm filter. Dopamine levels were analyzed using the HPLC column (3 mm × 150 mm, C-18 reverse phase column, Atlantis T3 3 µm, Thermo Scientific) with a dual channel coulochem III electrochemical detector (Model 5300, ESA Inc.). The 60 ng of 3,4-dihydroxybenzylamine (DHBA) was used as an internal standard. The values were normalized to protein concentrations measured from a BCA protein assay kit (Pierce) and the data were expressed in ng/mg protein.

### Mass Spectrometry Sample Preparation.

Primary cortical neurons were preincubated with Irisin (50 ng/mL) for 1 h and α-syn PFF was administered the neurons were further incubated with Irisin for 1 or 4 d. Soluble lysates were extracted from cells using a buffer comprised of 1% Triton X-100 in Tris buffer (50 mM Tris, 150 mM NaCl, pH 7.4) and protease inhibitors. Protein concentration was measured and 15 μg of protein from each sample was prepared for MS analysis. Samples were diluted with an equal volume of the buffer (400 mM EPPS pH 8.5, 0.5% SDS, 10 mM Tris(2-carboxyethyl)phosphine hydrochloride) and incubated for 10 min at room temperature. Iodoacetimide (final concentration of 10 mM) was added and further incubated for 25 min in the dark, followed by DTT (final concentration of 10 mM) was added. A buffer exchange was carried out using a modified SP3 protocol as previously reported (43, 44). Briefly, ∼250 μg of each SpeedBead magnetic carboxylate modified particles (Cytiva; 45152105050250, 65152105050250) mixed at a 1:1 ratio were added to each sample. Samples were combined with ethanol to make a final ethanol concentration of at least 50% and incubated for 15 min with gentle shaking. After three washes washing with 80% ethanol, proteins were eluted from SP3 beads using 200 mM EPPS (pH 8.5) containing trypsin (ThermoFisher Scientific) and Lys-C (Wako) and digested overnight at 37 °C with vigorous shaking. Samples were combined with acetonitrile (final concentration of 33%) and then labeled with TMTpro-18plex reagents (∼65 μg) (ThermoFisher Scientific). After confirmation of >97% labeling, excess TMTpro reagents were quenched by addition of hydroxylamine (final concentration of 0.3%). Acetonitrile was removed from the pooled samples by vacuum centrifugation for 1 h and acidified using formic acid. The peptides were de-salted using a Sep-Pak Vac 50 mg tC18 cartridge (Waters) and eluted in 70% acetonitrile, 1% formic acid. Dried peptides were resuspended in 10 mM ammonium bicarbonate (pH 8.0) and 5% acetonitrile. Twenty-four fractions were collected after fractionation by basic pH reverse phase HPLC were dried, resuspended in 5% acetonitrile and 1% formic acid, and de-salted by stage-tip. The peptides were eluted in 70% acetonitrile and 1% formic acid, dried, and finally resuspended in 5% acetonitrile and 5% formic acid. A total 11 of 24 fractions were analyzed by LC-MS/MS.

### Mass Spectrometry Data Acquisition.

Data were acquired on an Orbitrap Eclipse mass spectrometer paired with a Proxeon EASY nLC 1000 LC pump. Prepared peptides were solubilized in 5% ACN/5% formic acid, loaded onto a C18 column (30 cm, 2.6 μm Accucore, 100 μm ID), and eluted over a 120-min gradient. High-field asymmetric-waveform ion mobility spectroscopy was used during data collection with compensation voltages (CVs) of −40 V, −60 V, and −80 V. MS1 precursor scans were acquired in the orbitrap with the following parameters: 120 K resolution, 4e5 AGC target, and a maximum of 50-ms injection time. The ion trap was used to collect MS2 scans (1 s per CV) using collisional induced dissociation fragmentation. MS2 scans were collected with the following settings: NCE 35%, 2e4 AGC target, maximum injection time 50 ms, isolation window 0.5 Da. Orbiter, a real-time search algorithm, was used to trigger MS3 quantification scans. These scans were acquired in the orbitrap with the following settings: 50,000 resolution, AGC of 2 × 10^5^–5 × 10^5^, injection time of 150 ms, HCD collision energy of 45%. Protein-level closeout was set to five peptides per protein per fraction for six fractions and two peptides per protein per fraction for five fractions (45).

### Mass Spectrometry Data Analysis.

Raw files were converted to mzXML. Monocle was used to reassign monoisotopic peaks (46). Database searching used all mouse entries from Uniprot (July 2014) combined with all protein sequences in the reversed order. The sequences of frequent contaminant proteins were also included. Comet was used to perform the searches using a 50-ppm precursor ion tolerance and 1.0005 Da product ion tolerance. Static modifications were set as follows: TMTpro on lysine residues and peptide N termini (+304.2071 Da) and carbamidomethylation of cysteine residues (+57.0215 Da). Methionine oxidation (+15.9949 Da) was set as a variable modification.

Peptide-spectrum matches were filtered to a 1% false discovery rate (FDR) (47) using linear discriminant analysis (LDA) on each run as described previously (48). LDA used the following parameters: comet log expect, different sequence delta comet log expect (percent difference between the first hit and the next hit with a different peptide sequence), missed cleavages, length of peptide, charge state of peptide, mass accuracy of the precursor, and percentage of ions matched. In contrast to peptide-level FDR, which filtered each run separately, protein-level FDR was estimated at the level of the full dataset. For each protein, the posterior probabilities (as determined by LDA) for an individual peptide were multiplied to yield a protein-level probability estimate. Proteins were filtered to the target 1% FDR level, utilizing the Picked FDR method (49).

In order to quantify reporter ions, a 0.003 Da window around the theoretical *m/z* of each reporter ion was scanned, using the most intense *m/z*. Reporter ion intensities were corrected for the isotopic impurities using specifications provided by the manufacturer. Only peptides with a summed signal-to-noise (across all channels) greater than 160 were included. TMTpro signal-to-noise values for individual peptides were summed to quantify proteins.

### Statistical Analysis or Proteomic Data.

Statistical analysis was performed using Perseus (50). Total quantified proteins were filtered to remove unreviewed TrREMBL sequences and proteins quantified using a single peptide. A permutation-based FDR was used to identify significant changes. The following settings within Perseus were used: FDR, 0.05; S0, 0.1; and number of randomizations, 250.

### Statistical Analysis.

Data are from at least three independent biological replicates. The graphs are represented as mean ± SEM with statistical analysis using GraphPad Prism 7 software. Differences between two groups were analyzed with unpaired two-tailed Student *t* test and differences among multiple groups were analyzed by ANOVA followed by Tukey’s post hoc test. *P* < 0.05 was considered statistically significant.

## Supplementary Material

Supplementary File

Supplementary File

## Data Availability

The mass spectrometry data were deposited to the ProteomeXchange Consortium (PXD032670) (51). Correspondence and requests for materials and access to datasets should be addressed to B.M.S or T.M.D. All study data are included in the article and/or *SI Appendix*.
