# Supplementary material for: Amelioration of pathologic α-synuclein-induced Parkinson’s disease by irisin
Source: Proc Natl Acad Sci U S A. 2022 Aug 31;119(36):e2204835119. doi: 10.1073/pnas.2204835119 (PMC9457183; doi:10.1073/pnas.2204835119)
Supplement: Supplementary File [file pnas.2204835119.sapp.pdf]

PNAS
www.pnas.org

# Supplementary Information for
Amelioration of Pathologic $\alpha$-Synuclein-Induced Parkinson's Disease by Irisin

Tae-In Kam, Hyejin Park, Shih-Ching Chou, Jonathan G. Van Vranken, Melanie J. Mittenbuhler, Hyeonwoo Kim, Mu A, Yu Ree Choi, Devanik Biswas, Justin Wang, Yu Shin, Alexis Loder, Senthilkumar S. Karuppagounder, Christiane D. Wrann, Valina L. Dawson, Bruce M. Spiegelman, Ted M. Dawson

Correspondence to: Bruce M. Spiegelman (bruce_spiegelman@dfci.harvard.edu)
or Ted M. Dawson, M.D., Ph.D. (tdawson@jhmi.edu)

**This PDF file includes:**

Figures S1 to S4
Table S1
Legend for Datasets S1

**Other supplementary materials for this manuscript include the following:**

None

## Supplementary Figure legends

### Supplementary Figure 1

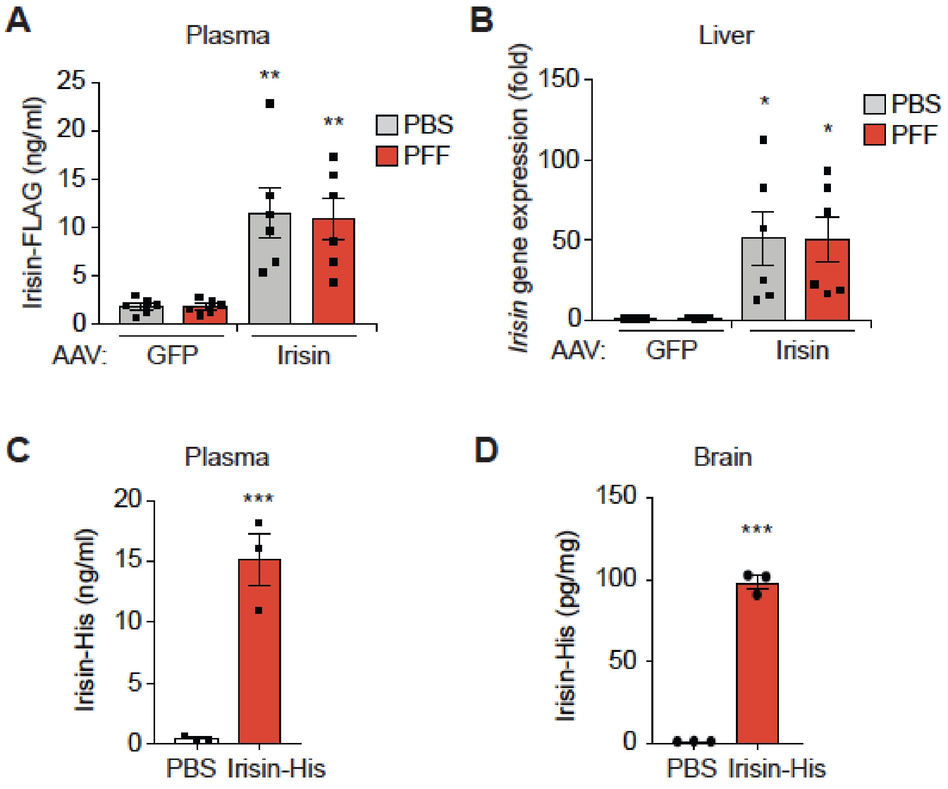


**Figure S1. Blood-brain penetration of intravenously injected irisin.**

**(A, B)** Two weeks after intrastriatal α-syn PFF injection, the mice were injected with AAV8-GFP or AAV8-Irisin-FLAG (1E10 G.C./mouse) via the tail vein. Six months after α-syn PFF injection, (A) irisin-FLAG levels in the plasma and (B) *irisin* mRNA expression in the liver were determined by ELISA and qPCR, respectively. Bars represent mean ± s.e.m. Two-way ANOVA followed by Tukey's post hoc test. (n=6 mice per group).

**(C, D)** C57BL/6 mice were intravenously (IV) injected with 1 mg/kg of purified irisin-His for 1 h. The concentration of irisin in (C) plasma and (D) brain were measured by ELISA. Bars represent mean ± s.e.m. Two-way ANOVA followed by Tukey's post hoc test. (n=3 mice per group). *$P$ < 0.05, **$P$ < 0.005, ***$P$ < 0.0005.

## Supplementary Figure 2

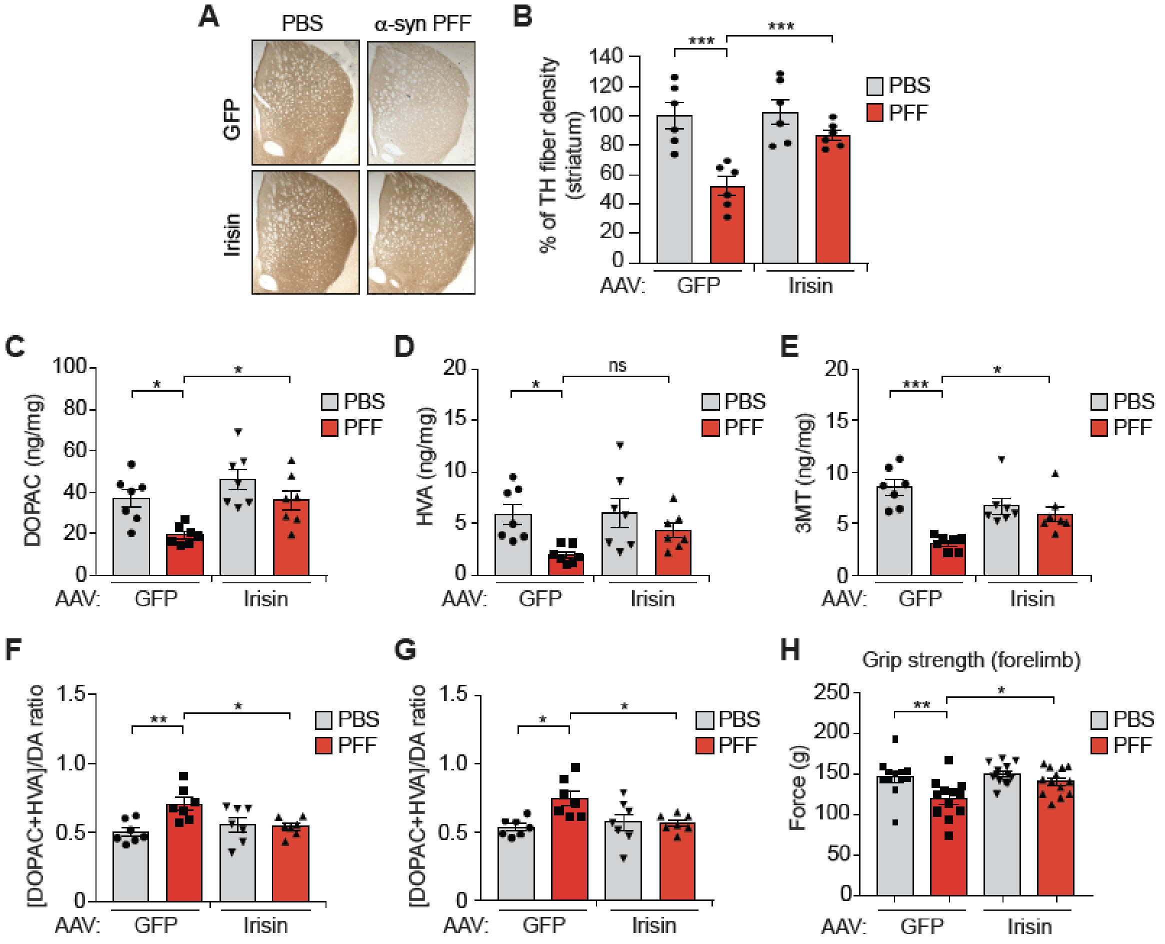


**Figure S2. Irisin protects α-syn PFF-induced pathology in vivo.**

**(A)** Representative photomicrograph of striatal sections stained for TH fiber density.

**(B)** Quantification of dopaminergic fiber densities in the striatum using Image J software (NIH). Bars represent mean ± s.e.m. Two-way ANOVA followed by Tukey's post hoc test (n=6).

**(C-E)** (C) DOPAC, (D) HVA and (E) 3MT concentrations in the striatum of PBS or α-syn PFF injected mice treated with AAV-GFP or AAV-Irisin at 6 months after α-syn PFF or PBS injection

measured by HPLC. Bars represent mean ± s.e.m. Two-way ANOVA followed by Tukey's post hoc test. (n=7 mice per group).

**(F, G)** DA turnover as determined by (F) (DOPAC+HVA)/DA and (G) (DOPAC+3MT)/DA was calculated from the striatum. Bars represent mean ± s.e.m. Two-way ANOVA followed by Tukey's post hoc test (n=7 mice per group).

**(H)** 180 days after intrastriatal α-syn PBS or PFF injection, grip strength test were performed. Data are the mean ± s.e.m. Two-way ANOVA followed by Tukey's post hoc test (n=12-13 mice per group). *$P < 0.05$, **$P < 0.005$, ***$P < 0.0005$.

## Supplementary Figure 3

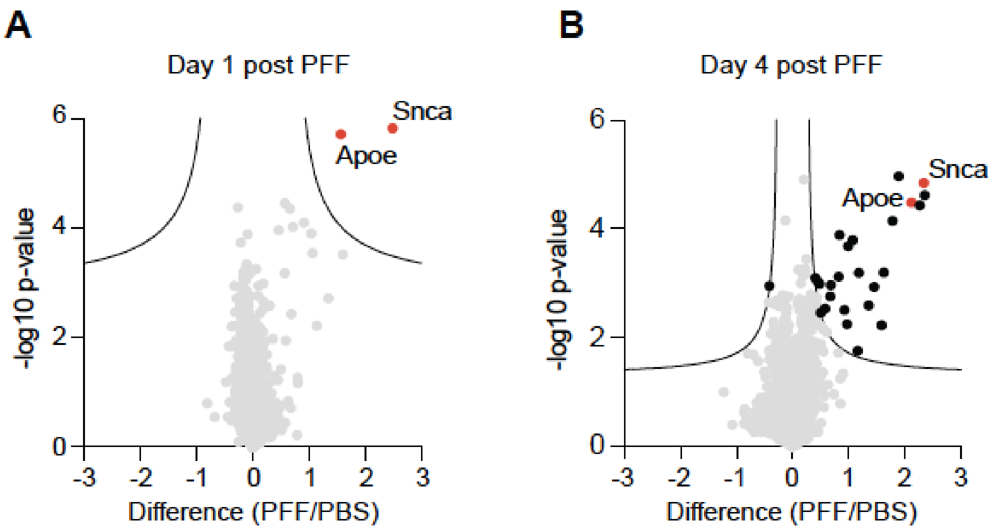


**Figure S3. Tandem mass spectrometry analysis of α-syn PFF-treated neurons.**

**(A, B)** Volcano plot of protein alterations. The proteins quantified from primary cortical neurons treated with PBS or α-syn PFF (1 μg/ml) for (A) 1 or (B) 4 days were analyzed for differentially expressed proteins in α-syn PFF treated cells.

# Supplementary Figure 4

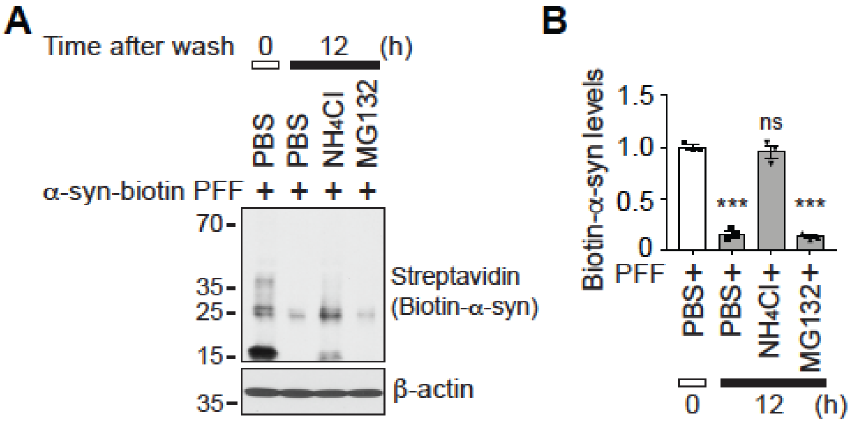


**Figure S4. Propagated α-syn PFF degraded by lysosome.**

**(A, B)** Primary cortical neurons pretreated with PBS, NH4Cl or MG132 were further incubated with biotin-conjugated α-syn PFF (1 μg/ml) for 12 h. Twelve hours after changing to fresh medium, the intracellular biotin-conjugated α-syn PFF levels were determined by immunoblotting using anti-streptavidin antibody. Bars represent mean ± s.e.m. One-way ANOVA followed by Tukey's post hoc test (n=3). ***$P$ < 0.0005.

## Supplementary Tables

**Table S1. The list of antibodies used in this study**

| Antibodies | Source | Identifier | Dilution |
|---|---|---|---|
| α-syn | BD Bioscience | 610787 | 1:1,000 (WB) |
| p-α-syn (ser129) | Biolegend | 825701 | 1:500 (IF) |
| p-α-syn (ser129) | Cell Signaling | 23706 | 1:1,000 (WB) |
| TH | Novus Biologicals | NB300-109 | 1:2,000 (WB)<br>1:1,000 (IHC) |
| DAT | Sigma | D6944 | 1:1,000 (WB) |
| Streptavidin-HRP | Thermo Fisher | N504 | 1:2,000 (WB) |
| Rab7 | Cell Signaling | 2094S | 1:1,000 (WB) |
| Lamp2 | Abcam | ab13524 | 1:1,000 (WB) |
| HSP60 | Cell Signaling | 12165 | 1:1,000 (WB) |
| β-actin-HRP | Sigma | A3854 | 1:20,000 (WB) |

**Supplementary Data Set S1. List of all the identified proteins with raw quantity values.**
